# Adherence to cardiovascular medication: a review of systematic reviews

**DOI:** 10.1093/pubmed/fdy088

**Published:** 2018-05-29

**Authors:** K H Leslie, C McCowan, J P Pell

**Affiliations:** 1 Institute of Health and Wellbeing, University of Glasgow, 1 Lilybank Gardens, Glasgow, UK; 2 Robertson Centre for Biostatistics, Institute of Health and Wellbeing, University of Glasgow, Boyd Orr Building, Glasgow, UK

**Keywords:** behaviour, circulatory disease, systematic review

## Abstract

**Background:**

Use of cardiovascular medication has increased over time, especially for primary and secondary prevention, with polypharmacy common.

**Methods:**

Review of published systematic reviews of the factors and outcomes associated with adherence to cardiovascular medication using MEDLINE, Embase, CINAHL and PsycINFO databases. Quality was assessed using the AMSTAR tool.

**Results:**

Of 789 systematic reviews identified, 45 met the inclusion criteria and passed the quality assessment; 34 focused on factors associated with adherence, and 11 on outcomes. High heterogeneity, both between and within reviews, precluded meta-analysis and so a pooled estimate of adherence levels could not be derived. Adherence was associated with disease factors, therapy factors, healthcare factors, patient factors and social factors, though with some inconsistencies. In total, 91% of reviews addressing outcomes reported that low adherence was associated with poorer clinical and economic endpoints.

**Conclusions:**

Factors from across five key domains relate to non-adherence to cardiovascular medications, and may contribute to poorer clinical outcomes. Interventions to improve adherence should be developed to address modifiable factors and targeted at those at highest risk of non-adherence. Adherence research is highly heterogeneous to-date and efforts to standardize this should be implemented to improve comparability.

## Introduction

Cardiovascular disease (CVD) is the leading cause of death globally.^1^ Due to an ageing population and proliferation of clinical trials, use of cardiovascular medication and polypharmacy have increased over time, which may contribute to non-adherence to drugs. The US National Health and Nutrition Survey (NHANES) found that 77% of adults diagnosed with hypertension had been prescribed an antihypertensive drug in 2010, compared to 63% in 2001.^2^ Additionally, the incidence of polypharmacy has risen, with the percentage of patients taking multiple antihypertensives in the US NHANES cohort growing by 11%.^2^ Within the UK, the percentage of adults aged 65–84 years who are prescribed three or more medications for chronic conditions has increased by 50.5%,^3^ while for those aged over 85 it has increased by 21.6%.^3^ This, in turn, has led to increased costs to health services: NHS England dispensed over 1000 million prescriptions in 2015, at an increase of 16.8% in costs from 2005, totalling £9267 million for net ingredient expenditure.^4^

Trial evidence of efficacy will only translate into real-world effectiveness if levels of adherence achieved in research studies can be replicated in the general population. In addition to disease management, cardiovascular medications are used in both primary and secondary prevention. Adherence may be particularly problematic when medication is used as a long-term, preventive strategy rather than for symptom relief.^4^ Patients’ perception of the risk associated with their disease may also play a role; for example, adherence to HIV medication has been shown to be 5% higher than adherence to CVD medication.^5,6^ The aim of this study was to review the existing published evidence of the factors and outcomes associated with adherence to CVD medications.

## Methods

Searches were conducted using MEDLINE (1996–present), Embase (1996–present), CINAHL and PsycINFO (full search strategy, Appendix 1). Inclusion was restricted to systematic reviews written in English, and duplicate publications removed using EndNote X7. Titles, then abstracts, then full text were reviewed manually and included if they studied factors associated with adherence to CVD medication for management of symptoms, primary, or secondary prevention, or the association between adherence and health outcomes. Reviews that included other conditions, as well as CVD, were included but those focused exclusively on non-CVD medication were excluded. Publications were excluded if they focused on interventions to improve adherence, adherence to non-medical interventions such as behavioural change, or guidelines on management of adherence. Data extraction included information on study aims, setting, methods, search strategies and findings (overall adherence levels and differences between sub-groups where reported). Review of papers and data extraction was performed by K.L., with a 10% sample independently reviewed by C.M., Findings were compared and disagreements discussed to reach consensus.

The quality of eligible studies was assessed using the AMSTAR tool,^7^ with papers scored out of 11 and categorized into high quality (score of 9–11), reasonable quality (score 6–8) or poor quality (score 3–5). Papers that scored ≤2 were rejected.

## Results

**Fig. 1:**
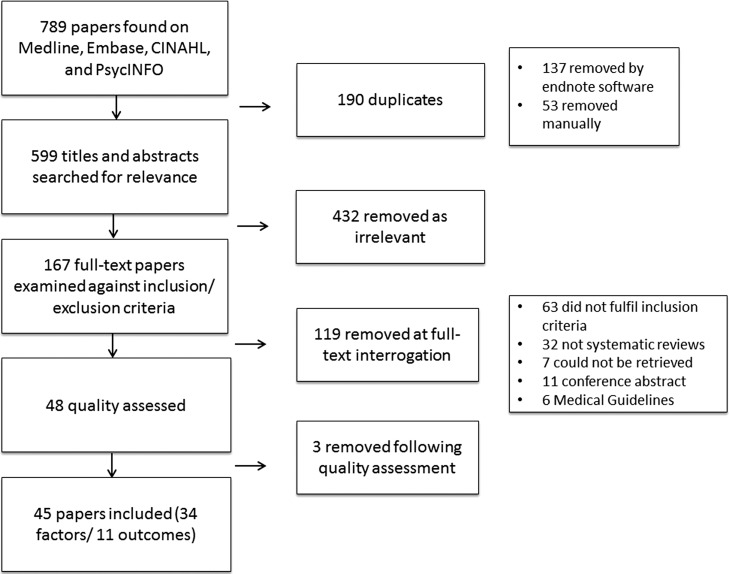
Summary flowchart of paper selection.

The literature search initially identified 789 papers. After review (741 papers removed) and quality assessment (3 removed) there were 45 eligible systematic reviews, 34 of which dealt with factors associated with non-adherence and 11 on outcomes (Fig. 1).

Most reviews scored as high quality (*n* = 16, 36%), or reasonable quality (*n* = 20, 44%) using the AMSTAR tool, but many noted that the primary studies included were of a variable standard. Results from data extraction of eligible reviews are summarized in Supplementary Tables. Most primary studies were conducted in the USA, Europe, and other developed countries, with only two systematic reviews centred on low or middle income countries (LMICS).^8,9^ Half of the systematic reviews reported an overall estimate of adherence, though this often had a wide range within each review, the most extreme example ranging from 20 to 88%.^10^ Most systematic reviews included studies employing a range of methods to study adherence, with self-reporting most common, followed by pharmacy claims, prescription refills and pill counts. Electronic monitoring methods were reported in fewer reviews (*n* = 11), possibly due to increased costs associated with. Measures of adherence also varied, with many studies comparing ‘good’ versus ‘bad’ adherence across a specified threshold (≥0%). The two most common adherence measures are the Medical Possession Ratio (MPR), the ‘number of days covered with medication in the refill gap, divided by the number of days in the refill gap’,^11^ and Proportion of Days Covered (PDC), or the ‘number of days with medication supplied divided by the length of follow-up’.^11^ Others considered the relative change in adherence rates between groups, or the hazard ratio for non-adherence against a reference category, though most reviews failed to specify which of these metrics was used in the primary studies.

Overall, 34 systematic reviews studied factors associated with adherence,^5,6,8–39^ broadly categorized into: disease factors, therapy factors, healthcare factors, patient factors and social factors^6,40^ (Table 1). Due to heterogeneity in study design, quality, and operational definitions of adherence, it was not possible to perform meta-analysis to derive a pooled risk estimate associated with any individual factor. Instead, detailed summaries of key factors are provided in Table 2.

**Table 1:** Factors found to impact adherence

Disease factors	Therapy factors	Healthcare factors	Patient factors	Social factors
Disease treated^5^	Side-effects^8,12,13^	Relationship/communication with physician^6,14,15^	Gender^9,16,17^	Socioeconomic status^6,10,17,19–21^
Primary versus secondary disease prevention^11,17,20,22^	Dosing regimen/frequency^5,8,22–24^	Self-monitoring^25^	Age^5,17^	Level of education/health literacy^9,26^
Comorbidity^10^	Drug class^10,12,13,27,28^	Cost/co-payments^6,8,20,22,29–31^	Making time for appointments^6,29^	Ethnicity^16^
Depression^10,14,32^	Combination pill^33–37^	Routine place of care^6,30^	Stress/anxiety^14^	Minority status^10^
Diabetes^10,17^		Routine physician^6,15,30^	Forgetfulness^6,14^	Social support^18^
Duration of treatment^20^		Practitioner disagreement with guidelines^14^	Lack of understanding^8,14,15,29^	
		Coronary artery calcium (CAC) screening^39^	Alcohol consumption^39^	
			Patient beliefs/perception of drugs^6,8,14,15,21^

**Table 2:** Consistency of findings across reviews for key adherence factors

Factors	Reviews	Findings*	Consistency
Disease			
Primary versus secondary	Mann *et al.* 2010; Lemstra *et al.* 2012; Xu *et al.* 2016; Chen *et al.* 2015	Secondary + Primary −	Similar findings across 100% of reviews. Of 10 studies comparing adherence or persistence following MI to primary, 90% found better adherence in MI group, with one study non-significant.^17^ Primary group 52% less likely to adhere than secondary across 18 studies.^20^ Overall adherence 46% better for secondary prevention compared with primary prevention population at 1 year (PDC).^22^ Increased adherence following hospitalization in all studies^11^ and trend continues with number of subsequent hospitalizations.^11^
Comorbidity- diabetes	Mann *et al.* 2010; Lemstra and Alsabbagh 2014	Diabetes +	Similar findings across both reviews, though variation at study level. 54% Agreement across studies,^17^ with the remaining studies non-significant, and one found diabetes comorbidity negatively impacted adherence. Review found 85% agreement across studies.^10^
Comorbidity- depression	Khatib *et al.* 2014; Lemstra and Alsabbagh 2014; Eze-Nliam *et al.* 2010	Depression −	Depression common barrier, identified in 42% of self-reported studies.^14^ Meta-analysis of five studies (eight cohorts) found 11% increased risk of nonadherence if depressed or prescribed anti-depressants.^10^ Only one cohort found inconsistencies.^10^ Of two studies looking at depression, one found reduced adherence and the other found a non-significant odd ratio.^32^
Therapy			
Drug Class	Matchar *et al.* 2008; Powers *et al.* 2012; Lemstra and Alsabbagh 2014; Kronish *et al.* 2011; Bramlage *et al.* 2009	ARB’s + ACEi’s +/− CCB’s +/− BB’s +/− Diuretics –	Found overall adherence was >90% for both ACEi and ARBs. In 11 studies of persistence all found higher continuation rate in ARBs (absolute difference of 7% between groups).^12^ Whether or not this difference is significant is not stated. 39 Studies of adherence or persistence. Nine studies which utilized pill counts found over 90% adherence to ARBs or ACEi’s (no significant difference) and persistence best with ARBs compared to ACEi’s.^13^ High level of agreement between studies: diuretics had an increased risk of non-adherence compared with ACEi’s (RR 1.36), ARB’s (RR1.47), CCB’s (RR1.35).^10^ Pooled analysis found higher risk of non-adherence in ACEi’s (RR1.30) and CCB’s (RR1.33) compared to ARBs.^10^ Across 17 studies, ARB’s consistently associated with better adherence (non-significant result in one study).^27^ Similarly diuretics found to be associated with poorer adherence in all but two studies—one non-significant result and another favoured diuretics compared to BB’s.^27^ ARB’s highest persistence in 87% of studies. Next best persistence found in ACEi’s, CCB’s, and then BBs. Diuretics lowest adherence rate in 100% of studies.^28^
Dosing frequency/Treatment Regimen	Assawasuwannakit *et al.* 2015; Bowry *et al.* 2011; Iskedjian *et al.* 2002; Ingersoll *et al.* 2008	High dosing freq. – High complexity +/−	Largely consistent, as number of dosages per day increases adherence decreases. As age increased, the reduction in adherence associated with dosing frequency became less significant.^5^ Divided into two subgroups: over 50% of patients take two or more drugs per day and group where less than 50% fall into this category. Found no significant difference in adherence levels between subgroups.^8^ Largely consistent across eight studies. Pooled results found average adherence of 91% for once-daily, which was 8% higher than for multiple daily dosing.^23^ Once daily was also higher compared to twice daily dosing (5% better for once daily).^23^ The difference in adherence rate between twice and multiple daily dosing groups was not significant. Across six studies assessing dosing regimen, adherence was best with once daily dosing compared to ≥2 per day in all but one study.^24^ Disease may play a role—the study which was not consistent looked specifically at patients with congestive heart failure while other studies looked at hypertension or CVD more generally.
Healthcare			
Cost	Bowry *et al.* 2011; AlGhurair *et al.* 2012; Lemstra *et al.* 2012; Xu *et al.* 2016; Marshall *et al.* 2012; Maimaris *et al.* 2013, Mann *et al.* 2014	Higher costs −	82% of studies found significant association between high medication cost and non-adherence.^8^ Financial burden and medication reimbursement cited as reasons for patient non-adherence in seven self-reported studies.^6^ Pooled result across six studies found that patients liable for co-payments are 28% less likely to be adherent.^20^ Cost/co-payment most commonly studied factor (29% studies).^22^ Nine studies (seven US, two Brazil) found cost cited as a barrier to non-adherence.^29^ 14 Studies investigated medication costs or co-payments; seven cohort studies found lower adherence with higher costs, with one exception which found increased adherence at much higher co-payment levels. Remaining cross-sectional and case-control studies also found higher adherence associated with lower costs.^30^ Variable results across six studies. Some found greater levels of significance than others, though this related to how much patients had to pay (i.e. less significant if cost lower—backs up principle).One study inconsistent—found that higher co-payment reduced level of adherence, while all other found opposite.^31^
Patient			
Gender	Lewey *et al.* 2013; Mann *et al.* 2010; Nielsen *et al.* 2017	Female gender +/-	Inconsistent. Varied with setting. 10% less likely to be adherent in females compared to males across 53 studies.^16^ 20% (*n* = 11) of studies found no difference between genders, all conducted in Canada.^16^ Female gender associated with lower adherence in 61% studies.^17^ In four studies carried out in LMIC’s women were less likely to adhere than men (OR 0.72).^9^
Age	Assawasuwannakit *et al.* 2015; Mann *et al.* 2010	Increasing age +/−	Significant association between age and adherence in a 77% of studies, though not linear – adherence improved with ages up to 65 yrs, then declined in older adults.^5^ Studies which did not observe this tended to have an older study cohort and hence only found the decline in adherence associated with older age (over 65).^5^ Review only found improvement—8% increase in adherence per 10 years increase in age, though maximum age included was 66.7 years.^17^
Perceptions	Bowry *et al.* 2011; Khatib *et al.* 2014; Rashtid *et al.* 2014; AlGhurair *et al.* 2012; McKenzie *et al.* 2015.	Perceive ill health +/− Perception of reduction in symptoms − Perceive drugs as addictive or harmful −	Negative perception of medication common barrier reported in 52% of studies, 73% of which found statistical significance.^8^ 30% of self-reported studies looking at patient factors found patient perception of medications as a barrier.^14^ Pooled: in 10 self-reported studies, perceptions about consequences were reported as a barrier in 19% of surveys.^15^ Across 17 qualitative studies, found that patients with either no/reduced symptoms or those with very severe symptoms less likely to adhere as they believe they are not ill or that it is futile as their disease has already progressed too far.^6^ Suggests patient perception may be a strong barrier to adherence though gives no quantitative summary.^21^
Social			
SES	AlGhurair *et al.* 2012; Mann *et al.* 2010; Alsabbagh *et al.* 2014; Lemstra *et al.* 2012; McKenzie *et al.* 2015; Lemstra and Alsabbagh 2014.	Higher SES + Lower SES −	In 22% of self-reported studies socioeconomic factors were cited as reducing adherence.^6^ Overall found that those with a higher income more likely to adhere,^17^ though inconsistent at study level—55% found this effect while 44% found no difference between low and high income groups.^17^ Of 32 studies 17 found higher income associated with higher adherence, 14 were non-significant, while 1 found lower adherence.^19^ In 11 studies, odds of adhering improve with higher income.^20^ Lower income (concession card holders in Australia healthcare system) found to have higher adherence. May be confounded (co-payments from concession card may improve adherence).^21^ Pooled analysis across nine studies found overall lower adherence with lower income status.^10^

*Improved adherence (+), decreased adherence (−) or inconsistent (+/−) relationship with adherence.

### Disease factors

Whilst adherence to medication for secondary prevention following acute coronary syndrome was suboptimal,^11^ it was nonetheless greater than adherence for primary prevention.^17,20,22^ The effect of comorbidity on adherence varied according to the condition. Patients with diabetes had higher adherence to CVD medications,^10,17^ while depression almost universally had a negative impact on adherence.^10,14,32^ The duration of treatment was also important, with adherence tending to decline over time.^20^

### Therapy factors

In spite of heterogeneity in specific study characteristics,^27^ drug class was consistently associated with differences in adherence.^10,12,13,27,28^ Adherence was best with angiotensin-II receptor blockers (ARB’s),^10,12,13,27,28^ and in pooled results, those prescribed ARB’s were 30–33% more likely to be adherent overall compared to those prescribed other drug classes.^10,27^ Diuretics were associated with the lowest adherence rates^10,27^ and lower persistence rates of any drug class, ranging from 16 to 38% across studies,^28^ compared to beta-blockers, BB’s (26–50%), calcium-channel blockers, CCB’s (26–52%), angiotensin converting enzyme inhibitors, ACEi’s (28–64%), and ARB’s (26–68%).^28^ Differences in side-effects may partly explain these variations. Patients on ACEi’s are 68% more likely to develop a cough than those on ARB’s.^12^ At standard dose, side-effects are more prevalent among patients taking thiazides (occurred in 9.9%), BB’s (7.5%) and CCB’s (8.3%), compared with ACEi’s (3.8%),^28^ while ARB’s (0%) are not associated with any side-effects.^28^

Dosage and treatment regimen were also associated with adherence. Combination drugs were associated with greater adherence compared to equivalent drugs given separately, ranging from 12% in one meta-analysis,^33^ to 29% in another.^34^ Overall, adherence declined as the number of doses per day increased,^5,22–24^ however, this effect was reduced with increased age.^5^ In LMIC’s, approximately half of studies looking at dosing complexity found a significant relationship between >1 daily dosing and non-adherence.^8^ Of five reviews that examined treatment regimens, two did not take account of concomitant drug use as a factor,^8,22^ and a further two acknowledged this but stated they were unable to analyse it given the data available.^5,23^ Only Ingersoll *et al.*^24^ discussed polypharmacy; 66% of studies of CVD medication found a positive influence of polypharmacy on adherence, despite increasing complexity being found to have a reductive effect on adherence elsewhere. The third study produced inconsistent findings.

### Healthcare factors

Medication or appointments costs to the patient was commonly identified as influencing non-adherence,^6,8,20,22,29–31^ largely in US settings. One review reported cost or co-payment as the most commonly studied factor related to adherence (29% of studies).^22^ Patients who had to make co-payments for treatment were at a 28% greater risk of non-adherence, as they were less likely to collect their statins at the appropriate time,^20^ and similar associations were found across cohort studies of adherence to antihypertensives.^30^ The impact of co-payment on adherence varied depending on the actual cost to the patient.^31^

Continuity of care was found to positively influence adherence.^6,15,30^ In one review,^30^ nine of eleven studies reported that it had a positive influence on patient awareness of their condition, treatment, or control of hypertension.^30^

### Patient factors

Gender was studied in three reviews.^9,16,17^ The majority of studies identified an association between gender and nonadherence, with a 7–10% increased risk of non-adherence among women. However, no association with gender was reported in any of the eleven studies conducted in Canada^16^ and, in studies conducted in low- and middle-income countries, adherence was better among women.^9^ Age was also associated with adherence: pooled results by Assawasuwannakit *et al.*^5^ demonstrated a 9% improvement in adherence over a 13-year increase in age, from 40 to 53 years, among patients with hypertension, however, no data was included for patients over the age of 67 (age range: 6.9–66.7 years).^5^ Mann *et al.*^17^ found a ‘u-shaped’ relationship between nonadherence and age, with middle aged patients having better adherence than adults aged between 18 and 50 years, or above 70 years old.

Other patient factors associated with poorer adherence include stress, anxiety and difficulty making time for appointments.^6,14,29^ Alcohol consumption has a negative affect on adherence^14,39^ across various chronic diseases, though findings are inconsistent^39^ with insufficient research specific to hypertension.^39^

In studies using patient self-reporting to measure adherence, forgetfulness and lack of knowledge were frequently cited risk factors^6,14^ and patient’s perception of medication and understanding of their disease were also important. In one review investigating qualitative patient self-reported studies (*n* = 15), three studies (20%) cited that patients discontinue treatment due to an initial lack of symptoms or following a reduction of symptoms,^14^ as they do not understand the chronicity of their disease. Perceptions about the medication itself can also have an impact; in the same review, two self-reported studies (13%) cited patient fears of reliance on cardiovascular drugs^14^ as a barrier to adherence.

### Social factors

The literature on socioeconomic status (SES) is inconsistent. In six systematic reviews reporting SES, two found no significant link^6,21^ although they did comment that other factors may have impacted this.^21^ In four reviews, high income status was associated with better adherence ranging from 11 to 26% across reviews^10,17,19,20^ though there was considerable variation at individual study level. In the review by Alsabbagh *et al.*,^19^ 77.5% of studies found a positive association between high SES and adherence, though all but one of the remaining studies found high SES had a negative impact on adherence.

Loke *et al.*^26^ investigated the relationship between health literacy and adherence to CVD or diabetes medications, but only one of seven cardiovascular studies found a significant association.

### Outcomes of non-adherence

Eleven papers^41–51^ reviewed studies of outcomes associated with non-adherence (Table 3), although the quality of these was lower than the reviews of risk factors; 64 and 85%, respectively, scoring reasonable or high quality. All but one study found a significant association between good adherence and improved clinical or economic outcomes; the exception of the Jongstra *et al.*^51^ review found no significant association between persistence of antihypertensive medication and cognitive function. Heterogeneity precluded meta-analysis.

**Table 3:** Outcomes associated with adherence

Outcome	References
Blood pressure control	^41,46,47,50^
Myocardial infarction	^46,48^
CVD risk	^42,45^
CVD deaths	^44,46,49^
All-cause mortality	^42,46^
Hospital admissions	^41,46^
Healthcare costs	^34^
Cognitive function	^51^

Bramlage and Hasford^28^ compared cost-effectiveness across drug classes, and found newer drugs, ARB’s and ACEi’s, outperformed the others in spite of being more expensive per tablet. This may be due to greater adherence to these drug classes^10,12,13,27,28^ which reduces later costs of CVD treatment and adverse events. Bitton *et al.*^43^ found that, in secondary prevention of CAD, patients who took ≤80% of their prescribed medication cost up to US$868 more per patient due to increased hospitalizations compared with the adherent group. Furthermore, Shroufi and Powles^49^ found improving adherence may reduce healthcare costs more than earlier prescribing of statins would do, highlighting this as an important aspect of disease management.

## Discussion

### Main finding of this study

Previous reviews have identified five main categories of factors associated with adherence; disease, therapy, healthcare, patient and social factors.^6,40^ As drugs taken for prevention of CVD are often intended for life-long use, adherence is especially important; recognizing and addressing factors that can improve adherence is vital in achieving maximum clinical and cost effectiveness. Side-effects and differences in adherence across drug classes could have important implications in prescribing of CVD drugs, while identifying different population sub-groups, such as those with co-morbid conditions, different genders, SESs or age groups, could be important in informing dosage regimes or targeting interventions to improve adherence.

There is lack of consensus around the association between adherence and outcomes, though nonetheless there is some evidence suggesting that better adherence leads to improved clinical and economic consequences, and there are gaps in the literature yet to be addressed.^47,48^ There was much heterogeneity across all studies with regards to how adherence rates were assessed.

From these findings, it is apparent that poor adherence to CVD medications has important consequences, and is a vital area of study in order to reduce CVD morbidity and mortality and maximize the cost-effectiveness of treatment.

### What is already known on this topic

It is well understood that adherence to drugs in chronic conditions is sub-optimal and there is a dearth of research into what the causative factors for this could be. However many studies are restricted in that they investigate single factors in isolation, or are of a relatively small scale and so have limited power. In terms of systematic reviews, there has been much research but little opportunity for meta-analysis owing to the huge heterogeneity existing within the literature.

### What this study adds

Many previous systematic reviews tend to focus on a particular factor, rather than looking across studies to include factors from each of the five groups; disease, therapy, healthcare, patient and social factors.^40^ This review collates all of this information into one place, to give an overview of factors identified throughout the literature. Few systematic reviews dealt with outcomes of nonadherence, which gives scope to develop research in this area.

Some factors were found to have similar associations with adherence across the literature, a good example being the class of drug prescribed. Newer CVD drugs, ARB’s and ACEi’s, were consistently found to have higher adherence rates than BB’s CCB’s, and diuretics.^10,12,13,27,28^

On the other hand, some factors had inconsistent relationships with adherence across primary studies and systematic reviews: for instance, female gender was associated with an overall higher risk of nonadherence in two reviews,^16,17^ though not elsewhere.^9^ It would be worthwhile identifying cultural, societal or policy differences that may influence this. Gender is a complex factor as it can be predictive of many other confounding factors, for example, woman more commonly assume a care-giving role than men,^16^ and this has been associated with poorer adherence.^16^ Hence, better support for caregivers and social care infrastructures could potentially help contribute to better adherence in this subgroup.

Many factors are likely to impact each other. Age will be influenced by different perceptions about health, increased comorbidities, and lifestyle changes. Generally, age was associated with an increase in adherence, though in the very old there is decline.^17^ This could be related to disease factors, for example, comorbidities common to old age, such as a declining cognitive function and therefore an increased likelihood to forget.^5^

Many perception barriers, such as belief that a reduction of symptoms indicates that drugs are no longer required,^14^ or concerns of dependence to CVD medications,^14^ illustrate a lack of understanding in some patients, and improved communication is required.

### Limitations of this study

Few systematic reviews were undertaken before the 1990s^52^ so it is unlikely that many papers would be missed by limiting searches to the 1996 version of the databases.

This review is limited in that it was restricted to papers written in the English language, contributing to potential publication bias. The huge levels of heterogeneity within systematic reviews included, and between them, made meta-analysis impossible and is a symptom of an area of research that has been largely unstandardized in its implementation. As this is an overview of systematic reviews, it would be impossible to perform meta-analysis without unpicking the individual studies to ensure none are over-represented. Another issue is that the study design and method for calculating adherence have both been found to alter the rates of adherence identified, though there is no gold standard within the literature for analysing this. Many systematic reviews failed to summarize operational definitions of adherence used by primary studies, i.e. whether studies looked at adherence as a continuous variable, or used a cut-off value above which individuals were considered adherent.

Performing a systematic review of reviews is a good way to collate and quality assess numerous studies published in this field; however, it is possible that important primary papers have been missed by focusing only on reviews.

## Conclusions

There is a range of modifiable and non-modifiable risk factors that have been associated with non-adherence to cardiovascular drugs, and these must be considered when developing interventions to improve disease management. Studies to-date are of variable quality and considerable heterogeneity. While some systematic reviews consider multiple factors, many primary studies look at risk factors in isolation, not accounting for the interplay between them, and because of heterogeneity there was no opportunity to study this quantitatively. This gives scope to conduct a primary study looking at multiple adherence factors from across these groups. Vitally important to this field of research is an agreed terminology and methodology, to allow comparisons across different study populations to be made. Vrijens *et al.*^53^ have defined a taxonomy recommended for use, and if used consistently by researchers it will greatly enhance the value of adherence research.

## Supplementary Material

Supplementary Data

Supplementary Table 1

Supplementary Table 2
