# Supplementary material for: Adherence to cardiovascular medication: a review of systematic reviews
Source: J Public Health (Oxf). 2018 May 29;41(1):e84–94. doi: 10.1093/pubmed/fdy088 (PMC6459362; doi:10.1093/pubmed/fdy088)
Supplement: Supplementary Data [file fdy088_appendix1_search_strategy.docx]

# Appendix 1: Search Strategy

## Search in Medline; Dates Jan 1996 - Jan 2017:

1 (adherence or compliance or non?adherence or non?compliance or persistence or non?persistence).ti,ab.

2 (hypertens* or antihypertens*).ti,ab.

3 ((cardiovascular* or CVD) and prevention).ti,ab.

4 2 or 3

5 (patient or medication* or drug or treatment).ti,ab.

6 1 and 4 and 5

7 limit 6 to (english language and humans and systematic reviews)

*All other bibliographic databases followed strategy as closely as possible, with minor modifications to cater for their specific requirements.*

**Inclusion Criteria**

Systematic Reviews of:

- Factors associated with adherence to CVD medication (used for management of symptoms, primary or secondary prevention)

- OR the association between adherence to CVD medication and health outcomes.

- Reviews that included other conditions, as well as CVD, were included

**Exclusion Criteria**

- focused on interventions to improve adherence

- adherence to non-medical interventions (such as behavioural change)

- guidelines on the management of adherence

- Not a systematic review

- reviews that focused exclusively on non-CVD conditions

- Papers that scored <=2 on quality assessment with AMSTAR tool

- Conference abstracts (with no paper associated that could be retrieved)

- Papers could not be accessed
